# Chilaiditi's Syndrome: An Unusual Cause of Acute Respiratory Distress

**DOI:** 10.1002/ccr3.71491

**Published:** 2025-11-18

**Authors:** Prosper Adjei, Kingsley Owusu Manu, Paa Kwesi Asante Addison, Emmanuel Ntow, Bismark Kyeremeh, Basit Tordia

**Affiliations:** ^1^ Department of Internal Medicine Methodist Hospital Wenchi Ghana

**Keywords:** Chilaiditi's sign, Chilaiditi's syndrome, respiratory distress, shortness of breath

## Abstract

Chilaiditi's syndrome is characterized by hepatodiaphragmatic interposition of the bowel, usually colon, with associated symptoms. Occasionally, affected individuals may experience acute respiratory distress and retrosternal pain which can mimic life‐threatening conditions like pulmonary embolism and myocardial infarction. This case report underscores the need for clinicians to be aware of this rare syndrome as it is amenable to conservative treatment in most instances.

## 1 Introduction

Chilaiditi's syndrome is a rare condition characterized by the interposition of the colon between the liver and the right hemidiaphragm, causing symptoms [1]. It is also referred to as symptomatic hepatodiaphragmatic interposition of the colon [2]. The bowel segments commonly involved in this condition are the hepatic flexure and proximal transverse colon. Occasionally, trapping of the small bowel may occur [3, 4].

The occurrence of bowel interposition between the liver and the right hemidiaphragm without associated symptoms is termed as Chilaiditi's sign [4]. This radiological abnormality which was first described in 1910 by Demetrius Chilaiditi [5] is often discovered incidentally [3]. Its incidence ranges from 0.025% to 0.28% on abdominal or chest radiographs. The estimated incidence on abdominal computed tomography (CT) scan is 1.18% to 2.4% [6].

There are several etiological factors of Chilaiditi's syndrome. Some of these factors are hepatic in origin and include liver cirrhosis, hepatectomy and laxity of the falciform ligament. Dolichocolon, elongation of the suspensory ligaments of the transverse colon and phrenic nerve palsy also predispose individuals to developing Chilaiditi's syndrome. Other factors implicated in hepatodiaphragmatic interposition of the colon are ascites, obesity and post‐operative intra‐abdominal adhesions [4, 7, 8].

Chilaiditi's syndrome is usually associated with gastrointestinal symptoms such as abdominal pain, abdominal distension, bloating, constipation, anorexia, nausea and vomiting. Rarely, affected individuals may complain of respiratory distress and retrosternal pain [9]. This case report describes an elderly man who had acute onset shortness of breath while on admission in our hospital, and was eventually found to have Chilaiditi's syndrome.

## 2 Case History and Examination

A 70‐year‐old man, who was admitted to our hospital and was receiving treatment for right knee septic arthritis, had sudden onset difficulty in breathing on the second day of admission. There was no associated chest pain, chest tightness, heaviness in the chest, cough, hemoptysis, fever, orthopnea, paroxysmal nocturnal dyspnea or bipedal swelling. He also complained of constipation which he had for 3 days, but was able to pass flatus. He did not have abdominal distension, abdominal pain, nausea or vomiting. He was ambulant and not bedridden at home. He denied a previous medical history of hypertension, ischemic heart disease, diabetes mellitus, asthma or any other chronic illness. He had never had any surgeries in the past. He was a non‐alcoholic who did not use illicit drugs or smoke cigarettes. The significant finding from his admission notes was a swollen and moderately tender right knee with differential warmth, and mild restriction of active and passive range of motion. Systemic examination at initial presentation was unremarkable. His medications included intravenous clindamycin 600 mg 8 hourly, intravenous paracetamol 1 g three times daily, and subcutaneous enoxaparin 40 mg daily.

Upon reassessment, he was acutely ill‐looking, dyspneic, afebrile (36.3°C), anicteric, not pale, not cyanosed, not diaphoretic, and had no pedal edema or unilateral leg swelling. His oxygen saturation was 58% on room air. He was alert and oriented to person, place, and time. He had a regular pulse with a rate of 95 beats per minute and blood pressure of 136/78 mmHg. Jugular venous pressure was not elevated and precordial examination was normal. His respiratory rate was 28 cycles per minute with reduced air entry in the right lower lung zone, vesicular breath sounds, and minimal fine crackles in the left lower lung zone on chest auscultation. His abdomen moved with respiration. It was full and soft with no guarding or rebound tenderness. There were no palpable organs and bowel sounds were normal. The calculated clinical pretest probability of pulmonary embolism was low (i.e., modified Wells score = 0).

## 3 Investigations, Diagnosis, and Treatment

**FIGURE 1:**
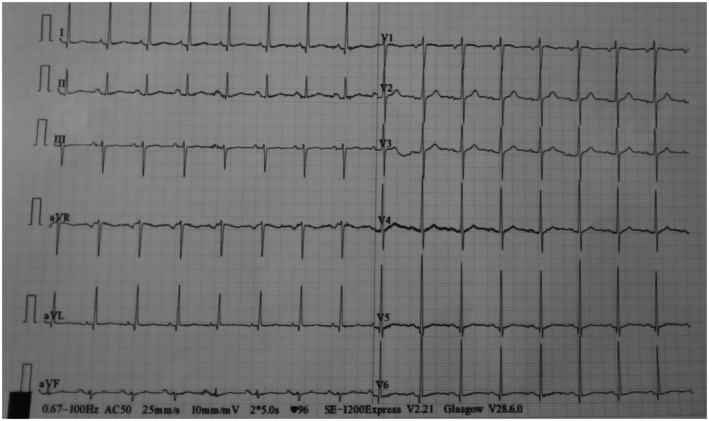
Resting 12‐lead ECG showing normal sinus rhythm with no ST‐segment or T wave changes.

**FIGURE 2:**
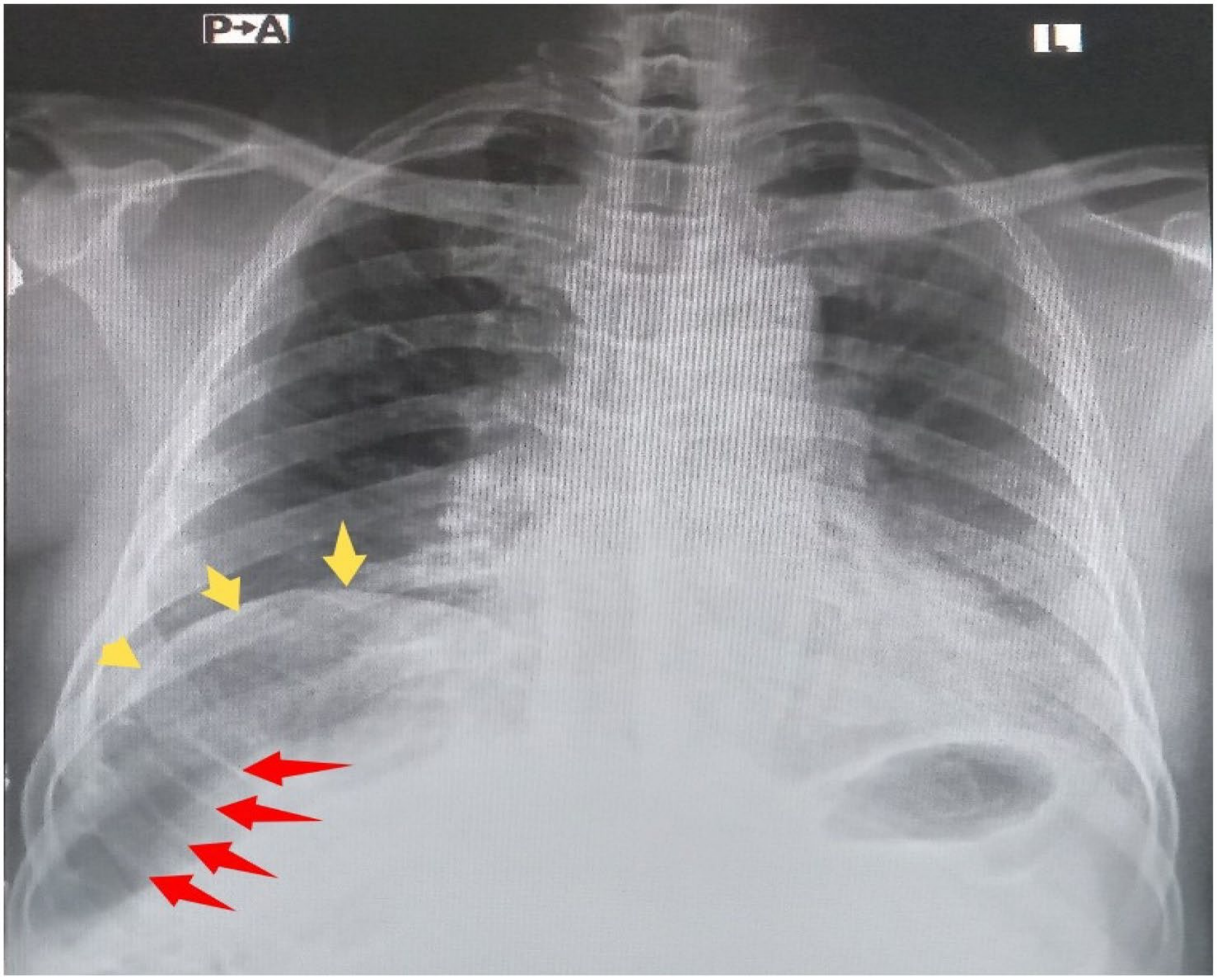
Posteroanterior chest x‐ray showing elevation of the right hemidiaphragm (yellow arrow) and a large volume of air with haustral folds (red arrow) below the diaphragm.

Oxygen supplementation via nasal prongs at 5 L per minute was immediately started following which his saturation rose to 93%. An urgent 12‐lead electrocardiogram (ECG) showed normal sinus rhythm with no ST‐segment or T‐wave changes (Figure 1). Cardiac enzymes and serum D‐dimer levels were normal (Table 1). Also, full blood count, renal function, serum electrolytes, and liver biochemistries were within normal range. Posteroanterior chest x‐ray revealed elevation of the right hemidiaphragm with a large volume of air beneath it. There were haustral folds within the air under the right hemidiaphragm. Also noted was inferior displacement of the liver below the level of the left hemidiaphragm (Figure 2). Abdominal ultrasound scan was normal. The patient could not afford an abdominal CT scan due to financial constraints.

**TABLE 1:** Relevant laboratory results for the patient.

Laboratory parameter	Patient's result	Reference range
Cardiac enzymes		
Troponin I (ng/mL)	0.10	< 0.30
Troponin T (ng/mL)	0.02	< 0.10
Creatine kinase MB (U/L)	9.2	< 24
D‐dimer (mg/L)	0.20	0.0–0.50

Based on the above, a diagnosis of Chilaiditi's syndrome was made. He was managed conservatively with bed rest, intravenous fluids, and lactulose 15 mL three times daily. The other medications for the septic arthritis were continued.

## 4 Outcome and Follow‐Up

**FIGURE 3:**
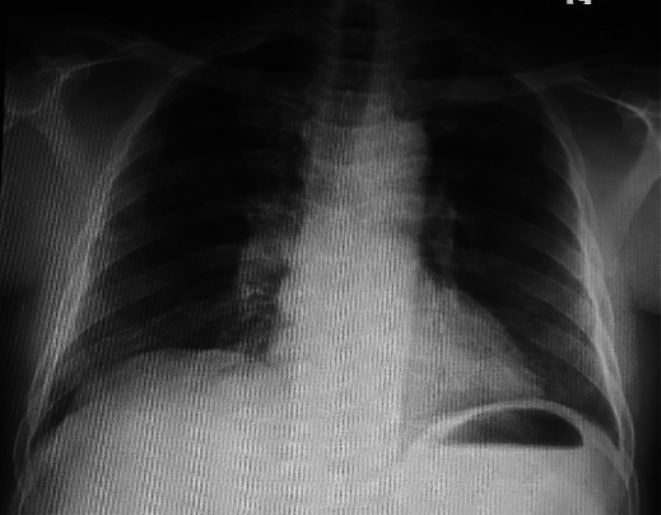
A repeat posteroanterior chest x‐ray showing complete resolution of the air under the right hemidiaphragm.

He was able to pass stools the next day. The dyspnea resolved completely after 72 h of conservative treatment and he was accordingly weaned off oxygen. A repeat chest x‐ray showed complete disappearance of the air under the right hemidiaphragm (Figure 3). He was eventually discharged after 10 days of hospitalization. At 1‐month follow‐up at the outpatient clinic, he was doing well with no recurrence of his symptoms.

## 5 Discussion

Chilaiditi's syndrome predominantly affects males with a male‐to‐female ratio of 4:1 [6]. Additionally, patients over 60 years of age are frequently affected [3]. Our patient is 70 years old and falls within this vulnerable age population. Chilaiditi's syndrome is commonly associated with gastrointestinal symptoms. Respiratory distress and retrosternal pain are however, infrequently encountered in patients with hepatodiaphragmatic interposition of the colon [9]. The interposed loop of bowel can exert pressure on the diaphragm, thereby reducing lung ventilation with resultant respiratory distress [1]. Few cases of Chilaiditi's syndrome causing acute respiratory distress have been reported in the literature.

Although it is generally considered a benign condition [10], the occurrence of acute respiratory distress or retrosternal pain in Chilaiditi's syndrome may mimic sinister conditions like pulmonary embolism and myocardial infarction. Given the sudden nature of our patient's presentation, it was critically important to exclude these life‐threatening conditions by doing an urgent 12‐lead ECG, cardiac enzymes and D‐dimer, all of which turned out to be normal. Chilaiditi's syndrome and Chilaiditi's sign share the same radiological features which comprise elevation of the right hemidiaphragm, air‐distended bowel illustrating pseudopneumoperitoneum, and inferior displacement of the liver below the level of the left hemidiaphragm [6]. The appearance of haustral folds within the air under the right hemidiaphragm as was observed in our case indicates that, the air is in the lumen of the interposed loop of bowel and not free [4]. The difference between Chilaiditi's syndrome and Chilaiditi's sign lies solely in the presence or absence of associated clinical symptoms. It must be emphasized that meticulous evaluation of the patient's clinical manifestations, as well as radiological features, is crucial for making a clear distinction between the former (symptomatic) and the latter (asymptomatic). Our patient's chest radiograph demonstrated the aforementioned radiological findings. These radiological criteria, without accompanying signs of peritonism, strongly suggested that Chilaiditi's syndrome was the most likely cause of the patient's acute respiratory distress. In addition, the complete resolution of the acute respiratory distress and chest radiograph findings following the initiation of conservative treatment further supported Chilaiditi's syndrome as the likely etiology of the patient's symptoms.

Conservative approach is the mainstay of treatment for Chilaiditi's syndrome. This involves bed rest, intravenous fluids, bowel decompression and laxatives [11]. In instances where the conservative approach fails, surgical intervention is indicated. Occasionally, Chilaiditi's syndrome may be complicated by bowel perforation, volvulus of the colon, mesenteric ischemia, and bowel obstruction. The development of these severe complications often warrants emergency surgical intervention [11]. It is worth noting that, accurate diagnosis and timely initiation of appropriate treatment can prevent or minimize the risk of complications.

In a similar reported case, a 66‐year‐old woman with cervical squamous cell carcinoma had sudden onset dyspnea and right‐sided chest pain on the second day after surgery. Her oxygen saturation rapidly dropped to 75% with an increased serum D‐dimer level. An emergency CT pulmonary angiography was performed which showed a trapped loop of colon between the liver and the right hemidiaphragm. There was no angiographic evidence of pulmonary embolism. Consequently, she was diagnosed with Chilaiditi's syndrome and treated with potassium supplementation and bowel decompression [12]. Even though our patient was receiving enoxaparin for thromboprophylaxis prior to developing acute respiratory distress, it was still necessary to rule out pulmonary embolism as prophylactic anticoagulation does not completely eliminate the risk of venous thromboembolism. In this case, the exclusion of pulmonary embolism was based on low clinical pretest probability (i.e., modified Wells score = 0) and normal D‐dimer level. The combination of low clinical pretest probability and normal or negative D‐dimer is a widely accepted and reliable method for ruling out pulmonary embolism [13]. This obviated the need for further imaging with CT pulmonary angiography in our patient.

In another report by Silva et al., a 78‐year‐old man developed acute onset shortness of breath which was associated with confusion, abdominal discomfort and constipation. His oxygen saturation was 89%. Chest radiograph and abdominal CT scan showed a loop of colon between the liver and the diaphragm. He was treated conservatively with gradual improvement in his symptoms [1]. Unlike the case described by Silva et al., our patient had a very low oxygen saturation (i.e., 58%) which improved significantly after oxygen supplementation. Again, the only gastrointestinal symptom our patient had was constipation.

Also, an elderly female reported at the emergency unit of a hospital with complaints of rapid onset difficulty in breathing and abdominal pain. CT scan of the abdomen revealed interposed loops of colon between the liver and the right hemidiaphragm. Her D‐dimer was within the normal range and she also responded favorably to conservative treatment just like our patient [14].

## 6 Limitation

An abdominal CT scan could not be performed because the patient was financially constrained.

## 7 Conclusion

Chilaiditi's syndrome is a rare benign condition which is often associated with gastrointestinal symptoms. Less commonly, individuals with this condition may experience acute respiratory distress and retrosternal pain which can mimic life‐threatening conditions like pulmonary embolism and myocardial infarction. It is therefore, critically important for clinicians to be aware of this rare syndrome as it is amenable to conservative treatment in most instances.

## Author Contributions

**Prosper Adjei:** conceptualization, data curation, investigation, writing – original draft, writing – review and editing. **Kingsley Owusu Manu:** conceptualization, data curation. **Paa Kwesi Asante Addison:** data curation. **Emmanuel Ntow:** data curation. **Bismark Kyeremeh:** data curation. **Basit Tordia:** data curation.

## Ethics Statement

The authors have nothing to report.

## Consent

Written informed consent was obtained from the patient to publish this report in accordance with the journal's patient consent policy.

## Conflicts of Interest

The authors declare no conflicts of interest.

## Data Availability

The authors have nothing to report.
